# Are Michigan State University medical school (MSU-CHM) alumni more likely to practice in the region of their graduate medical education primary care program compared to non-MSU-CHM alumni?

**DOI:** 10.1186/s12909-018-1225-z

**Published:** 2018-05-24

**Authors:** Richard Switzer, Luke VandeZande, Alan T. Davis, Tracy J. Koehler

**Affiliations:** 1 Spectrum Health/Michigan State University Internal Medicine Pediatrics Residency Program, 945 Ottawa Ave. NW, Grand Rapids, MI 49503 USA; 2 Spectrum Health OME Scholarly Activity Support, 945 Ottawa Ave. NW, Grand Rapids, MI 49503 USA; 3 Michigan State University Department of Surgery, 1200 E. Michigan Ave., Suite 655, Lansing, MI 48912 USA

**Keywords:** Primary care, Medical practice location, Fellowship, Proximity to GME training

## Abstract

**Background:**

Over the past 10 years, three new MD schools have been created in the state of Michigan, while the Michigan State University College of Human Medicine (MSU-CHM) has increased their class size to 850 students. The aim of this study was to determine if MSU-CHM alumni who graduate from an MSU-affiliated primary care residency from a single graduate medical education (GME) training program in Michigan are more likely to go on to practice in close proximity to the location of their training program immediately after graduation compared to non MSU-CHM alumni. Changes over time in the proportion of primary care graduates who received fellowship training immediately following residency were also compared between these groups.

**Methods:**

A review of historical data was performed for all 2000–2016 primary care (Family Medicine, FM; Internal Medicine, IM; Internal Medicine-Pediatrics, IMP; Pediatrics, Peds) program graduates sponsored by Grand Rapids Medical Education Partners (GRMEP). Study variables included primary care program, gender, age at graduation, fellowship training, practice location immediately after graduation and undergraduate medical education location. Summary statistics were calculated for the data. Comparisons were made using the chi-square test or Fisher’s Exact test when appropriate. Significance was assessed at *p* < 0.05.

**Results:**

There were 478 primary care program graduates who went into practice immediately following graduation, 102 of whom also graduated from MSU-CHM. Just over half of the graduates were female and the average age at graduation was 32 years. There were 152 FM, 150 IM, 50 IMP and 126 Peds graduates. Those that graduated from both MSU-CHM and GRMEP were more likely to practice in Michigan immediately after residency training (79.4% vs 52.0%; *p* < 0.001), as well as within 100 miles of GRMEP (71.6% vs 46.4%; *p* < 0.001). There were 8% of MSU-CHM primary care graduates who went on to fellowship training from 2000 to 2009, increasing to 34% from 2010 to 2016 (*p* < 0.001).

**Conclusion:**

Medical school graduates of MSU-CHM who receive GME training in primary care are more likely to practice medicine within close proximity to their training site than non MSU-CHM graduates. However, plans for fellowship after training may add one caveat to this finding.

## Background

In the early 2000’s, concerns were raised about a future shortage of physicians by the Council on Graduate Medical Education [1]. In response to this report, the American Association of Medical colleges recommended a 30% increase in enrollment of medical students, to be achieved by 2015 [2]. Through a combination of increased class sizes and an increase in the number of US medical schools, this goal has been met [3]. This increase has also been seen within the state of Michigan, where in the past 10 years, three new MD schools have been created (Central Michigan University College of Medicine, Oakland University William Beaumont School of Medicine, Western Michigan University School of Medicine), while the Michigan State University College of Human Medicine (MSU-CHM) has increased their class size from 400 to 850 students in two primary campuses, in East Lansing and in Grand Rapids [4]. This increase in class size was accompanied by the expansion of the preclinical campus to Grand Rapids in 2010.

The ultimate goal for the larger number of medical students was to grow the physician workforce, but as the number of residency positions has only slightly increased over the same time period as the medical school expansions, there have been some concerns raised as to whether or not augmenting the number of medical school graduates will actually meet national and regional needs for practicing physicians [5]. Koehler et al. have demonstrated that almost 80% of residency/fellowship graduates from a single GME training site in Michigan who also went to medical school in Michigan practiced medicine in Michigan [6]. MSU-CHM has long had a focus on the training of primary care physicians [7, 8], and Phillips et al. have shown that 44% of MSU-CHM graduates (from 1972 to 2006) were practicing as primary care physicians in 2011 [9]. Fagan et al. have shown that family medicine residency graduates are more likely to practice medicine in close proximity to their Graduate Medical Education (GME) training site [10]. However, the data are not currently available to show if MSU-CHM graduates, who enter MSU-affiliated primary care residency training programs in Michigan, are more likely to practice medicine in the same state or in close proximity to their training site following residency training, compared to non MSU-CHM graduates. An additional concern for primary care residency graduates is the increasing proportion of residency trained physicians who choose to receive subspecialty training following residency [3].

The primary objective of this study was to determine if MSU-CHM alumni who graduate from an MSU-affiliated primary care residency program from a single graduate medical education (GME) training program in Michigan are more likely to go on to practice in close proximity to the location of their training program immediately after graduation compared to non MSU-CHM alumni. A secondary objective was to look at the changes over time in the proportion of MSU-CHM primary care graduates who chose to receive further fellowship training immediately following their residency training.

## Methods

### Setting and study participants

A retrospective review of historical data was performed for all 2000–2016 primary care program graduates (Family Medicine, Internal Medicine, Internal Medicine-Pediatrics, Pediatrics) sponsored by Grand Rapids Medical Education Partners (GRMEP). During this time period, GRMEP sponsored MSU-affiliated training programs in Grand Rapids, Michigan, for just over 300 residents and fellows annually in various specialties and subspecialties. With regards to the first study objective, residents who graduated from one of the primary care training programs, who went immediately into a fellowship (i.e., subspecialty training program) or had a military obligation following graduation from GRMEP, were excluded from the review. The rationale for this exclusion was that our objective was to assess graduates who went into practice immediately following graduation. With regards to the second study objective, the only exclusion was for those graduates who were fulfilling a military obligation immediately following graduation from GRMEP. For the second objective, two time periods were used for comparison: 2000-2009 and 2010-2016.

### Data sources and study variables

Data were obtained from the New Innovations database (Uniontown, OH) and GRMEP GME department records of location after graduation data. Study variables included primary care program, gender, age at graduation, fellowship training, practice location immediately after graduation and undergraduate medical education location. The study was approved by the Spectrum Health Institutional Review Board.

### Objective one - Practice location following graduation

**Fig. 1:**
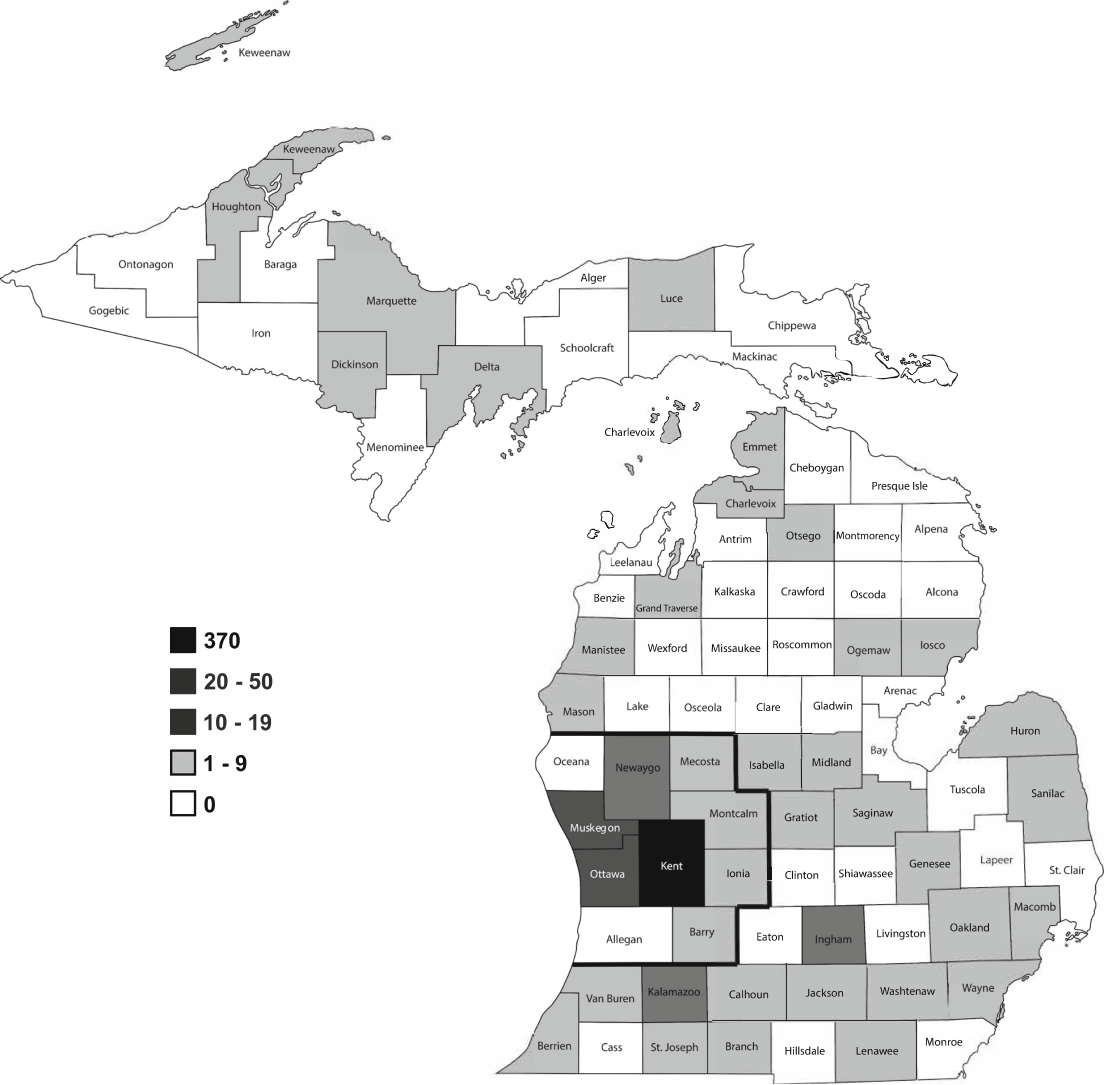
Practice locations of GRMEP GME Primary Care graduates by Michigan county. West Michigan counties are outlined in black. Graphic shows practice locations for all GRMEP GME Primary Care graduates from 2000 to 2016. The figure legend details the number of former residents/fellows practicing in each county. Image used under license from Shutterstock.com

This variable was examined in four different ways. The first was to compare primary care residents who graduated from MSU-CHM with those who graduated from other medical schools (US and foreign) with regard to practicing medicine in the state of Michigan immediately following graduation. The second was to compare the two groups with regard to practicing medicine in West Michigan immediately following graduation. This region was chosen, as GRMEP and the primary medical training sites are located in Grand Rapids in Kent County, in the Western part of the state. For the purposes of this study, West Michigan was defined as the counties of Allegan, Barry, Ionia, Kent, Mecosta, Montcalm, Muskegon, Newaygo, Oceana and Ottawa (Fig. 1). We also determined whether or not the primary care program residents who graduated from MSU-CHM and practice in Michigan were more likely to practice within 30 or 100 miles of Grand Rapids than those who received a medical degree from a different institution.

### Objective two – Proportion of primary care graduates entering fellowship programs

Two time periods were compared in this analysis, 2000 – 2009 and 2010 – 2016. The rates of primary care graduates who went on to a fellowship after graduation were compared between the two time periods.

### Analysis

Summary statistics were calculated for the data. Quantitative data are expressed as the mean and standard deviation, and nominal data are expressed as percentages. Comparisons were made using the chi-square test or Fisher’s Exact test when appropriate. Significance was assessed at *p* < 0.05. Analyses were performed using IBM SPSS Statistics v. 23 (Armonk, NY).

## Results

### Cohort demographics

There were 478 primary care program graduates who met our entry criteria for Objective 1, and 597 for Objective 2. Demographic and educational characteristics of each sample are shown in Table 1. Slightly less than half were male and the average age was 32 years old at the time of graduation. The majority completed medical school at colleges other than MSU-CHM. The breakdown by primary care residency program is also shown.

**Table 1:** Demographic and educational information

	Graduates excluding fellowship/military^a^ (*n* = 478)	Graduates excluding military^b^ (*n* = 597)
Age at graduation	32.4 ± 3.9^c^	32.4 ± 3.9^d^
Gender (male:female)	234:244	293:304
Primary Care Program		
Family Medicine	152 (31.8%)	161 (27.0%)
Internal Medicine	150 (31.4%)	197 (33.0%)
Internal Medicine-Pediatrics	50 (10.5%)	59 (9.9%)
Pediatrics	126 (26.4%)	180 (30.2%)
Medical School		
MSU-CHM	102 (21.3%)	125 (20.9%)
Other	376 (78.7%)	472 (79.1%)

*MSU-CHM* Michigan State University College of Human Medicine

^a^Primary care residency graduates, excluding those who entered a fellowship or military service immediately following graduation

^b^Primary care residency graduates, excluding those who entered military service immediately following graduation

^c^*n* = 477

^d^*n* = 596

### First study objective - Practice location immediately following graduation

**Fig. 2:**
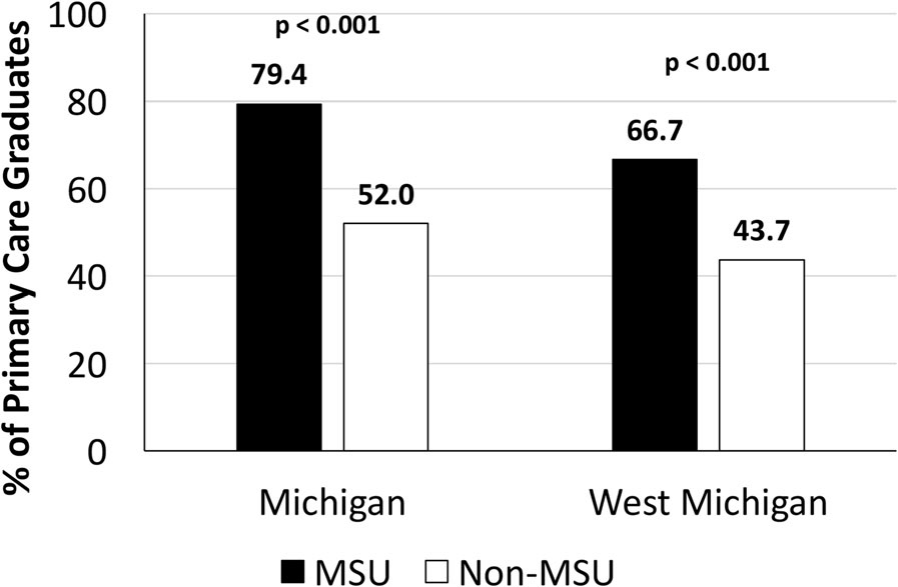
Practice locations of GRMEP GME primary care graduates immediately following graduation. Primary care graduates who underwent their medical school training at MSU-CHM were more likely to practice in Michigan and, more specifically, West Michigan after residency training, compared to non-MSU-CHM alumni (*p* < 0.001). Primary care was defined as Family Medicine, Internal Medicine, Internal Medicine-Pediatrics, and Pediatrics. West Michigan was defined as the following counties in Michigan: Allegan, Barry, Ionia, Kent, Mecosta, Montcalm, Muskegon, Newaygo, Oceana and Ottawa

**Fig. 3:**
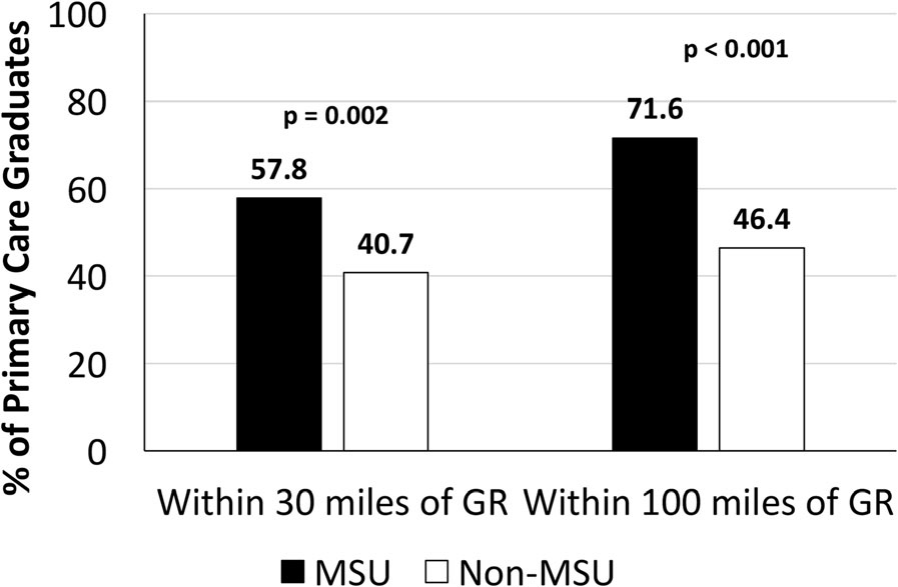
Proximity of practice locations of GRMEP GME primary care graduates immediately following graduation. GR: Grand Rapids, MI. Graduates who received their medical school training at MSU-CHM were significantly more likely to practice in Michigan within 30 miles (*p* = 0.002) or 100 miles (*p* < 0.001) of their GME training location, compared to non-MSU-CHM alumni. Primary care was defined as Family Medicine, Internal Medicine, Internal Medicine-Pediatrics, and Pediatrics

Figure 2 shows comparisons of practice location immediately following graduation between primary care residents who graduated from MSU-CHM and those that did not. MSU-CHM graduates were significantly more likely to practice in Michigan and in West Michigan (*p* < 0.001) immediately after residency training. They were also more likely to stay within 30 or 100 miles of Grand Rapids (Fig. 3).

Practice locations by the type of residency training program are shown in Table 2. A consistent finding was that MSU-CHM Family Medicine graduates were significantly more likely to practice medicine in close proximity to the site of their residency program, as opposed to non-MSU-CHM graduates. Additionally, MSU-CHM Internal Medicine graduates were more likely to practice medicine in the state of Michigan immediately following graduation.

**Table 2:** Practice location immediately following residency graduation^a^

	Practiced Medicine in Michigan Immediately Following Graduation		Practiced Medicine in West Michigan Immediately Following Graduation^b^	
	MSU-CHM^c^	Non-MSU^c^	MSU-CHM^c^	Non-MSU^c^
Family Medicine	23/23 (100%)^§^	61/129 (47.3%)^§^	21/23 (91.3%)^§^	47/128 (36.7%)^§^
Internal Medicine	16/19 (84.2%)^**^	73/131 (55.7%)^**^	11/19 (57.9%)^††^	65/130 (50.0%)^††^
Internal Medicine/Pediatrics	18/22 (81.8%)^††^	16/28 (57.1%)^††^	14/22 (63.6%)^††^	15/28 (53.6%)^††^
Pediatrics	24/38 (63.2%)^††^	45/87 (51.7%)^††^	22/38 (57.9%)^††^	36/87 (41.4%)^††^

*MSU-CHM* Michigan State University College of Human Medicine

^a^Excludes residents who entered a fellowship or military service immediately following graduation

^b^West Michigan includes the counties of Allegan, Barry, Ionia, Kent, Mecosta, Montcalm, Muskegon, Newaygo, Oceana and Ottawa

^c^Medical school attended prior to residency

^§^*p* < 0.001

^**^p = 0.018

^††^*p* > 0.05

MSU-CHM Family medicine graduates were also significantly more likely to practice medicine with 100 miles of GRMEP (22/23, 95.7%), as opposed to graduates from other medical schools (51/128, 39.8%; *p* < 0.001). There were no significant differences between MSU and non-MSU medical school primary care graduates when using a 100 mile limit for any of the other three residencies (data not shown).

### Second study objective – Proportion of primary care graduates entering fellowship programs

Table 3 shows fellowship trends by year groupings. There were 14.2% of primary care graduates who went on to fellowship training from 2000 to 2009. Almost twice as many went on to fellowship training from 2010 to 2016, which was a statistically significant increase. The results were even more striking for the MSU-CHM primary care graduates. Data for the individual primary care residency programs for all graduates are also shown in Table 3. The only individual program which showed a significant change was pediatrics, with almost a 2.5 fold increase in the later time frame, compared to 2000-2009 (*p* < 0.001).

**Table 3:** Percentage of residents who entered into a fellowship immediately following graduation from residency

	2000-2009	2010/2016	*p*-value
All primary care graduates	45/318 (14.2%)	74/279 (26.5%)	< 0.001
MSU-CHM primary care graduates^a^	6/75 (8.0%)	17/50 (34.0%)	< 0.001
Non-MSU-CHM primary care graduates^a^	39/243 (16.0%)	57/229 (24.9%)	0.017
All Family Medicine graduates	3/90 (3.3%)	6/71 (8.5%)	0.184
All Internal Medicine graduates	21/109 (19.3%)	26/88 (29.5%)	0.092
All Internal Medicine/Pediatrics graduates	6/32 (18.8%)	3/27 (11.1%)	0.488
All Pediatrics graduates	15/87 (17.2%)	39/93 (41.9%)	< 0.001

*MSU-CHM* Michigan State University College of Human Medicine

^a^Medical school attended prior to residency

## Discussion

Our results show that the majority of MSU-CHM graduates who completed a primary care residency at GRMEP and began their medical practice immediately following graduation did so within 30 miles of their training site. Furthermore, almost 80% of these primary care-trained physicians practiced medicine in the state of Michigan immediately following graduation. The results emphasize the value of GME programs in providing physicians to the local workforce, as well as to the state in which the program resides.

A subset of these data from GRMEP (2000-2014) had previously been used by Koehler et al. to report that medical school graduates from Michigan who went on to complete residencies within the state had a strong likelihood of practicing medicine in Michigan [6, 11]. These studies had been supported by national data, as well as data from South Carolina, Georgia, Virginia, New York, and Hawaii [12–18]. Koehler et al. developed a predictive model of in-state retention using data from GRMEP GME graduates, where they noted that completion of medical school in Michigan was a statistically significant predictor of the likelihood of practicing medicine in Michigan [11]. Kurahara et al. reported that Hawaiian Pediatrics residency graduates who were also graduates of Hawaii’s only medical school, the John A. Burns School of Medicine, were four times more likely to practice medicine in Hawaii following residency (based upon an odds ratio), compared to other medical school graduates [18]. The data from our study for all MSU-CHM primary care residency graduates are very similar to this report.

Fagan et al. noted that, in the US, almost 55% of Family Medicine graduates practice within 100 miles of their training program [10]. They further noted the percentage for graduates from 2000 to 2006 was even higher (62.5%). Our data from 2000 to 2016 showed that 66.7% of GRMEP primary care graduates practiced medicine in West Michigan, and that 49.1% of GRMEP Family Medicine graduates practiced within 100 miles of the training site immediately following graduation. This latter percentage increased to 95.7% when only MSU-CHM Family Medicine graduates were included.

While it is encouraging to note that the majority of MSU-CHM primary care residency graduates from our study chose to practice in Michigan immediately following graduation, it should be noted that the data excluded those who chose to go on to fellowship training after graduation, or those who had to fulfill a military obligation. Dalen and Ryan have reported an increase in the number of primary care program graduates who go on to fellowship training [3]. The effect was most marked for Pediatric residents, with 30% seeking subspecialty training in 2002, rising to 41% in 2015. This increase was also seen for internal medicine, rising from 62% of graduates to 81% of graduates in 2015. Family Medicine graduates who went on to subspecialty training remained constant at about 5% at both time points.

From our data, we saw a statistically significant increase from 2000 to 2009 compared to 2010-2016, with the percent of GRMEP primary care program graduates who went on to fellowships almost doubling from the earlier to the later time period. Even more striking was the significant rise in MSU-CHM primary care residency graduates, where a greater than four-fold increase in graduates going on to fellowships was seen, compared to the 1.5 fold increase seen for non-MSU-CHM primary care residency graduates. When looking at all GRMEP primary care residencies, the only significant increase was for Pediatrics, with a subspecialty training rate of 41.5% in 2010-2016, a value very similar to that reported by Dalen and Ryan for Pediatrics graduates [3]. While increases were also seen for GRMEP Family Medicine and Internal Medicine graduates, the findings were not statistically significant.

Interestingly, the GRMEP Internal Medicine/Pediatrics graduates actually showed a decrease in those going on for fellowship training. Our rates for 2000 – 2009 (18.8%) are similar to those reported by Chamberlin et al. for Internal Medicine/Pediatrics residents from 2003 to 2007 whose future practice goal was to enter fellowship training (18-26%) [19]. Our data imply an increased number of our Internal Medicine/Pediatrics graduates are going into primary care practice immediately following their residency training. However, no recent specific fellowship data are available for Internal Medicine/Pediatrics residents, so it is unclear whether the apparent decrease seen in our study relates to a national trend.

Limitations for this study include its retrospective nature, as well as the focus on the graduates from a single medical school in Michigan, whose graduates went on to GME training at a single residency training site, also within Michigan. In addition, for the testing of the second objective, a purely arbitrary decision was made to test the results from the first decade of the 2000’s against our 7 years of data from the second decade. Despite these limitations, it is clear that our study data have corroboration from other reports from the GME literature. Dalen and Ryan have shown an increase over time in the number of primary care trained residency graduates who go on to fellowship programs immediately following GME training [3]. In addition, the cut-point for the time frame chosen in our study for comparison (2000-2009 vs. 2010-2016) aligns with the year (2010) that MSU-CHM doubled the size of their medical school by expanding their campus in Grand Rapids, MI.

## Conclusions

In conclusion, our study supports the concept that medical school graduates from MSU-CHM who went on to complete their MSU-affiliated primary care residency education within the same state are more likely to practice medicine within that state, compared to non MSU-CHM medical school graduates. This has a major impact on physician workforce planning, and should be a consideration for residency programs, where one of the goals in recruitment is to select physicians most likely to remain in the community where they have trained. One caveat, however, is the increasing percentage of primary care graduates who are selecting fellowship training following graduation. A further look at this specific subset of GME graduates, particularly as it relates to where they practice medicine following their fellowship training, is warranted.

## Abbreviations

FM: Family medicine
GME: Graduate medical education
GRMEP: Grand Rapids Medical Education Partners
IM: Internal Medicine
IMP: Internal Medicine-Pediatrics
MSU-CHM: Michigan State University medical school
Peds: Pediatrics
US: United States

## References

[1] Council on Graduate Medical Education. Physician workforce policy guidelines for the United States, 2000-2020. 2005. https://www.hrsa.gov/advisorycommittees/bhpradvisory/cogme/Reports/sixteenthreport.pdf. Accessed 13 June 2017.

[2] American Association of Medical Colleges (2006). AAMC statement on the physician workforce.

[3] Dalen JE, Ryan KJ (2016). United States medical school expansion: impact on primary care. Am J Med.

[4] Michigan State University College of Human Medicine. Facts and Statistics. http://mdadmissions.msu.edu/Facts-stats/default.htm. Accessed 17 June 2017.

[5] Linville MD, Bates JE (2017). Graduate medical education – accelerated change. Am J Med Sci.

[6] Koehler TJ, Goodfellow J, Davis AT, vanSchagen JE, Schuh L (2016). Physician retention in the same state as residency training: data from 1 Michigan GME institution. J Grad Med Educ.

[7] Michigan State University College of Human Medicine Mission Goals and Objectives. East Lansing: Michigan State University College of Human Medicine; 1978.

[8] Mavis B, Sousa A, Osuch J, Arvidson C, Lipscomb W, Brady J, Green W, Rappley MD (2012). The College of Human Medicine at Michigan State University: expansion and reinvention. Acad Med.

[9] Phillips JP, Wendling AL, Fahey CA, Mavis BE (2018). The effect of a community-based medical school on the state and local physician workforce. Acad Med.

[10] Fagan EB, Gibbons C, Finnegan SC, Petterson S, Peterson LE, Phillips RL, Bazemore AW (2015). Family medicine graduate proximity to their site of training: policy options for improving the distribution of primary care. Access Fam Med.

[11] Koehler TJ, Goodfellow J, Davis AT, Spybrook J, vanSchagen JE, Schuh L (2017). Predicting in-state workforce retention after graduate medical education training. J Grad Med Educ.

[12] Burfield WB, Hough DE, Marder WD (1986). Location of medical education and choice of location of practice. J Med Educ.

[13] Seifer SD, Vranizan K, Grumbach K (1995). Graduate medical education and physician practice location: implications for physician workforce policy. JAMA.

[14] Office for Healthcare Workforce Analysis & Planning. Retaining Physicians Educated in South Carolina. 2011. https://www.scohw.org/docs/2011/Retaining-Physicians-Educated-in-SC.pdf. Accessed 13 June 2017.

[15] Georgia Statewide Area Health Care Network. 2013 Primary Care Summit Summary. Georgia Regents University website. http://www.gru.edu/ahec/documents/fy15gme_request.pdf. Accessed 13 June 2017.

[16] Owen JA, Hayden GF, Bowman RC (2005). Influence of places of birth, medical education, and residency training on the eventual practice locations of family physicians: recent experience in Virginia. Letter to the Editor. South Med J.

[17] Armstrong DP, Liu Y, Forte GJ. 2015 New York residency training outcomes: a summary of responses to the 2015 New York resident exit survey. Rensselaer: Center for Health Workforce Studies, School of Public Health, SUNY Albany; 2016. http://www.chwsny.org/our-work/reports-briefs/2015-new-york-residency-training-outcomes-a-summary-of-responses-to-the-2015-new-york-resident-exit-survey/. Accessed 13 June 2017

[18] Kurahara D, Yee K, Ifuku C, Herbst A, Deng D, Murai D, Rudoy R (2013). Medical school affects the career location of pediatric resident graduates. Hawai’i J Med Pub Health.

[19] Chamberlin JK, Frintner MP, Melgar TA, Kaelber DC, Correlates KBD (2012). Trends in training satisfaction on completion of internal medicine – pediatrics residency: a 5-year study. J Pediatr.

